# Supplementary material for: Modeling glioblastoma heterogeneity as a dynamic network of cell states
Source: Mol Syst Biol. 2021 Sep 16;17(9):e10105. doi: 10.15252/msb.202010105 (PMC8444284; doi:10.15252/msb.202010105)
Supplement: Supplementary file 5 — Source Data for Figure 3 [file MSB-17-e10105-s001.zip › Figure3A_sourcedata/GSEA_3065/nosigdb_state4.GseaPreranked.1622718430994/LARSSON_STATE_4.html]

Details for gene set LARSSON\_STATE\_4[GSEA]

|  || Dataset | state4 |
| Phenotype | NoPhenotypeAvailable |
| Upregulated in class | na\_neg |
| GeneSet | LARSSON\_STATE\_4 |
| Enrichment Score (ES) | -0.9240916 |
| Normalized Enrichment Score (NES) | -3.2877607 |
| Nominal p-value | 0.0 |
| FDR q-value | 0.0 |
| FWER p-Value | 0.0 |
Table: GSEA Results Summary

  

Fig 1: Enrichment plot: LARSSON\_STATE\_4      
 Profile of the Running ES Score & Positions of GeneSet Members on the Rank Ordered List

  

| PROBE | GENE SYMBOL | GENE\_TITLE | RANK IN GENE LIST | RANK METRIC SCORE | RUNNING ES | CORE ENRICHMENT || 1 | TUBA1B |  |  | 3658 | 0.007 | -0.5933 | No |
| 2 | LSM3 |  |  | 5075 | -0.089 | -0.8209 | No |
| 3 | H2AFV |  |  | 5381 | -0.117 | -0.8676 | No |
| 4 | TUBB |  |  | 5482 | -0.129 | -0.8807 | No |
| 5 | LSM4 |  |  | 5673 | -0.155 | -0.9078 | No |
| 6 | HNRNPD |  |  | 5705 | -0.161 | -0.9090 | No |
| 7 | NUCKS1 |  |  | 5724 | -0.166 | -0.9080 | No |
| 8 | SIVA1 |  |  | 5791 | -0.181 | -0.9144 | No |
| 9 | PTMA |  |  | 5852 | -0.195 | -0.9194 | Yes |
| 10 | HIST1H1E |  |  | 5865 | -0.198 | -0.9167 | Yes |
| 11 | ANP32B |  |  | 5890 | -0.204 | -0.9157 | Yes |
| 12 | SNRPD1 |  |  | 5911 | -0.210 | -0.9139 | Yes |
| 13 | LSM5 |  |  | 5932 | -0.216 | -0.9120 | Yes |
| 14 | RANBP1 |  |  | 5939 | -0.218 | -0.9078 | Yes |
| 15 | ALYREF |  |  | 5955 | -0.225 | -0.9049 | Yes |
| 16 | MND1 |  |  | 5982 | -0.232 | -0.9036 | Yes |
| 17 | RAN |  |  | 5990 | -0.235 | -0.8991 | Yes |
| 18 | STMN1 |  |  | 5993 | -0.236 | -0.8938 | Yes |
| 19 | RPA3 |  |  | 6014 | -0.244 | -0.8912 | Yes |
| 20 | HMGB1 |  |  | 6047 | -0.267 | -0.8900 | Yes |
| 21 | CDC45 |  |  | 6063 | -0.276 | -0.8859 | Yes |
| 22 | MCM5 |  |  | 6069 | -0.278 | -0.8800 | Yes |
| 23 | HIST1H1C |  |  | 6077 | -0.284 | -0.8744 | Yes |
| 24 | GMNN |  |  | 6080 | -0.285 | -0.8679 | Yes |
| 25 | FBXO5 |  |  | 6084 | -0.287 | -0.8616 | Yes |
| 26 | CENPN |  |  | 6090 | -0.291 | -0.8554 | Yes |
| 27 | DEK |  |  | 6092 | -0.292 | -0.8486 | Yes |
| 28 | RFC2 |  |  | 6095 | -0.294 | -0.8419 | Yes |
| 29 | DUT |  |  | 6107 | -0.303 | -0.8365 | Yes |
| 30 | MCM10 |  |  | 6110 | -0.305 | -0.8296 | Yes |
| 31 | MCM3 |  |  | 6113 | -0.307 | -0.8226 | Yes |
| 32 | PIMREG |  |  | 6115 | -0.310 | -0.8153 | Yes |
| 33 | ASF1B |  |  | 6127 | -0.320 | -0.8095 | Yes |
| 34 | PHF19 |  |  | 6129 | -0.322 | -0.8020 | Yes |
| 35 | LMNB1 |  |  | 6134 | -0.325 | -0.7949 | Yes |
| 36 | CENPH |  |  | 6136 | -0.327 | -0.7872 | Yes |
| 37 | TK1 |  |  | 6144 | -0.334 | -0.7804 | Yes |
| 38 | NUDT1 |  |  | 6146 | -0.338 | -0.7725 | Yes |
| 39 | RRM1 |  |  | 6147 | -0.338 | -0.7644 | Yes |
| 40 | E2F1 |  |  | 6152 | -0.343 | -0.7569 | Yes |
| 41 | KIF15 |  |  | 6158 | -0.346 | -0.7494 | Yes |
| 42 | EZH2 |  |  | 6159 | -0.348 | -0.7411 | Yes |
| 43 | CDKN3 |  |  | 6160 | -0.349 | -0.7328 | Yes |
| 44 | HMGN2 |  |  | 6163 | -0.351 | -0.7247 | Yes |
| 45 | KIF4A |  |  | 6164 | -0.355 | -0.7163 | Yes |
| 46 | RAD51AP1 |  |  | 6167 | -0.357 | -0.7081 | Yes |
| 47 | CENPK |  |  | 6169 | -0.363 | -0.6996 | Yes |
| 48 | MCM4 |  |  | 6171 | -0.372 | -0.6909 | Yes |
| 49 | PCNA |  |  | 6173 | -0.374 | -0.6821 | Yes |
| 50 | DTYMK |  |  | 6177 | -0.381 | -0.6735 | Yes |
| 51 | CENPM |  |  | 6179 | -0.386 | -0.6645 | Yes |
| 52 | DBF4 |  |  | 6180 | -0.387 | -0.6552 | Yes |
| 53 | CHAF1A |  |  | 6183 | -0.396 | -0.6461 | Yes |
| 54 | SGO1 |  |  | 6188 | -0.404 | -0.6371 | Yes |
| 55 | MYBL2 |  |  | 6190 | -0.408 | -0.6275 | Yes |
| 56 | SGO2 |  |  | 6192 | -0.413 | -0.6178 | Yes |
| 57 | GINS2 |  |  | 6194 | -0.420 | -0.6080 | Yes |
| 58 | ZWINT |  |  | 6195 | -0.425 | -0.5978 | Yes |
| 59 | BRCA1 |  |  | 6197 | -0.435 | -0.5876 | Yes |
| 60 | UHRF1 |  |  | 6198 | -0.436 | -0.5772 | Yes |
| 61 | RRM2 |  |  | 6199 | -0.438 | -0.5668 | Yes |
| 62 | DHFR |  |  | 6200 | -0.441 | -0.5563 | Yes |
| 63 | ESCO2 |  |  | 6206 | -0.463 | -0.5460 | Yes |
| 64 | PBK |  |  | 6207 | -0.465 | -0.5349 | Yes |
| 65 | FANCI |  |  | 6209 | -0.469 | -0.5239 | Yes |
| 66 | BIRC5 |  |  | 6214 | -0.484 | -0.5130 | Yes |
| 67 | HIST1H1A |  |  | 6216 | -0.487 | -0.5016 | Yes |
| 68 | GTSE1 |  |  | 6217 | -0.490 | -0.4899 | Yes |
| 69 | CLSPN |  |  | 6218 | -0.490 | -0.4782 | Yes |
| 70 | SMC4 |  |  | 6219 | -0.490 | -0.4665 | Yes |
| 71 | TYMS |  |  | 6222 | -0.501 | -0.4548 | Yes |
| 72 | NUSAP1 |  |  | 6223 | -0.507 | -0.4427 | Yes |
| 73 | NCAPG |  |  | 6227 | -0.513 | -0.4310 | Yes |
| 74 | SMC2 |  |  | 6229 | -0.518 | -0.4188 | Yes |
| 75 | TMPO |  |  | 6233 | -0.522 | -0.4068 | Yes |
| 76 | CKS1B |  |  | 6234 | -0.523 | -0.3943 | Yes |
| 77 | KPNA2 |  |  | 6236 | -0.543 | -0.3815 | Yes |
| 78 | H2AFX |  |  | 6237 | -0.576 | -0.3678 | Yes |
| 79 | ORC6 |  |  | 6238 | -0.585 | -0.3538 | Yes |
| 80 | CKS2 |  |  | 6239 | -0.585 | -0.3399 | Yes |
| 81 | UBE2T |  |  | 6240 | -0.593 | -0.3257 | Yes |
| 82 | HIST1H1D |  |  | 6241 | -0.601 | -0.3114 | Yes |
| 83 | CCNB1 |  |  | 6242 | -0.606 | -0.2969 | Yes |
| 84 | ATAD2 |  |  | 6243 | -0.607 | -0.2824 | Yes |
| 85 | UBE2S |  |  | 6244 | -0.617 | -0.2677 | Yes |
| 86 | PTTG1 |  |  | 6245 | -0.622 | -0.2529 | Yes |
| 87 | CDK1 |  |  | 6246 | -0.627 | -0.2379 | Yes |
| 88 | MAD2L1 |  |  | 6247 | -0.628 | -0.2229 | Yes |
| 89 | CENPU |  |  | 6248 | -0.633 | -0.2078 | Yes |
| 90 | HIST1H1B |  |  | 6249 | -0.646 | -0.1924 | Yes |
| 91 | H2AFZ |  |  | 6250 | -0.651 | -0.1769 | Yes |
| 92 | MCM7 |  |  | 6252 | -0.665 | -0.1612 | Yes |
| 93 | TPX2 |  |  | 6253 | -0.667 | -0.1453 | Yes |
| 94 | UBE2C |  |  | 6255 | -0.750 | -0.1275 | Yes |
| 95 | HMGB2 |  |  | 6258 | -0.766 | -0.1096 | Yes |
| 96 | PCLAF |  |  | 6259 | -0.790 | -0.0907 | Yes |
| 97 | TOP2A |  |  | 6260 | -0.814 | -0.0713 | Yes |
| 98 | MKI67 |  |  | 6261 | -0.882 | -0.0502 | Yes |
| 99 | HIST1H4C |  |  | 6262 | -1.038 | -0.0255 | Yes |
| 100 | CENPF |  |  | 6263 | -1.068 | -0.0000 | Yes |
Table: GSEA details [plain text format]

  

Fig 2: LARSSON\_STATE\_4: Random ES distribution      
 Gene set null distribution of ES for **LARSSON\_STATE\_4**
